# Etiological profile and treatment outcome of epistaxis at a tertiary care hospital in Northwestern Tanzania: a prospective review of 104 cases

**DOI:** 10.1186/1472-6815-11-8

**Published:** 2011-09-05

**Authors:** Japhet M Gilyoma, Phillipo L Chalya

**Affiliations:** 1 Department of Surgery, Weill- Bugando University College of Health Sciences, Mwanza, Tanzania

**Keywords:** Epistaxis, etiology, treatment outcome, Tanzania

## Abstract

**Background:**

Epistaxis is the commonest otolaryngological emergency affecting up to 60% of the population in their lifetime, with 6% requiring medical attention. There is paucity of published data regarding the management of epistaxis in Tanzania, especially the study area. This study was conducted to describe the etiological profile and treatment outcome of epistaxis at Bugando Medical Centre, a tertiary care hospital in Northwestern Tanzania.

**Methods:**

This was a prospective descriptive study of the cases of epistaxis managed at Bugando Medical Centre from January 2008 to December 2010. Data collected were analyzed using SPSS computer software version 15.

**Results:**

A total of 104 patients with epistaxis were studied. Males were affected twice more than the females (2.7:1). Their mean age was 32.24 ± 12.54 years (range 4 to 82 years). The modal age group was 31-40 years. The commonest cause of epistaxis was trauma (30.8%) followed by idiopathic (26.9%) and hypertension (17.3%). Anterior nasal bleeding was noted in majority of the patients (88.7%). Non surgical measures such as observation alone (40.4%) and anterior nasal packing (38.5%) were the main intervention methods in 98.1% of cases. Surgical measures mainly intranasal tumor resection was carried out in 1.9% of cases. Arterial ligation and endovascular embolization were not performed. Complication rate was 3.8%. The overall mean of hospital stay was 7.2 ± 1.6 days (range 1 to 24 days). Five patients died giving a mortality rate of 4.8%.

**Conclusion:**

Trauma resulting from road traffic crush (RTC) remains the most common etiological factor for epistaxis in our setting. Most cases were successfully managed with conservative (non-surgical) treatment alone and surgical intervention with its potential complications may not be necessary in most cases and should be the last resort. Reducing the incidence of trauma from RTC will reduce the incidence of emergency epistaxis in our centre.

## Background

Epistaxis or nasal bleeding is recognized as one of the most common otorhinolaryngological emergencies worldwide and presents a challenge in resource-poor centres where facilities for caring of these patients are limited [1]. Epistaxis is a problem frequently encountered in general practice and may present as an emergency, as a chronic problem of recurrent bleeds or may be a symptom of a generalized disorder [2]. It cannot only affect the hemodynamic but may cause great anxiety to patients and their relatives.

Epistaxis is estimated to occur in 60% of persons worldwide during their lifetime, and approximately 6% of those with nosebleeds seek medical treatment [1-4]. The prevalence is increased for children less than 10 years of age and then rises again after the age of 35 years [5]. Generally, males are slightly affected than females until the age of 50, but after 50 no deference between sexes as reported [2,4,5].

Epistaxis is commonly divided into anterior and posterior epistaxis, depending on the site of origin [5]. Anterior nosebleeds arise from damage to Kiesselbach's plexus on the lower portion of the anterior nasal septum, known as the Little's area, whereas posterior nosebleeds arise from damage to the posterior nasal septal artery [4,6]. Anterior epistaxis is far more common than posterior epistaxis, accounting for more than 80% of cases [4,6,7].

The aetiology of epistaxis can be broadly divided into the local or systemic causes, although even this distinction is difficult to make and the term "Idiopathic Epistaxis" is ultimately used in about 80-90% of the cases [4,8]. The etiological profile of epistaxis has been reported to vary with age and anatomical location [4-8]. Traumatic epistaxis is more common in younger individuals (under age 35 years) and is most often due to digital trauma, facial injury, or a foreign body in the nasal cavity [6-8]. Non-traumatic epistaxis is more characteristic of older patients (over age 50 years) and may be due to organ failure, neoplastic conditions, inflammation, or environmental factors (temperature, humidity, altitude) [7,8]. Epistaxis that occurs in children younger than 10 years usually is mild and originates in the anterior nose, whereas epistaxis that occurs in individuals older than 50 years is more likely to be severe and to originate posteriorly [9]. Epistaxis poses a greater risk in elderly people in whom clinical deterioration may progress rapidly if the blood loss is significant [7].

The treatment of epistaxis requires a systematic and methodical approach, and options vary according to the cause, location, and severity of the hemorrhage [4,6,7,9]. Both conservative and surgical treatment modalities have been used in the treatment of epistaxis [2,6]. However, their outcome has never been evaluated in our setting partly because of paucity of local data.

Most of the underlying causes of epistaxis are preventable [8,9]. A clearer understanding of the causes, treatment and outcome of these patients is essential for establishment of preventive strategies as well as treatment guidelines [1,7,8]. Such data is lacking in our environment as there is no local study which has been done on the subject. This study was conducted in our setting to identify the etiological profile and to determine the outcome of treatment of these patients. The results of this study will provide basis for planning of preventive strategies and establishment of treatment guidelines.

## Methods

### Study design and setting

This was a prospective descriptive study of patients who presented with nasal bleeding (epistaxis) at Bugando Medical Centre (BMC) over a three-year period from January 2008 to December 2010. BMC is a consultant, tertiary care and teaching hospital for Weill- Bugando University College of Health Sciences (WBUCHS) and has a bed capacity of 1000.

### Study subjects

The study subjects included all patients who presented with epistaxis at BMC during the period under study. These patients were received through Accident & Emergency department, ENT clinic and as referral from other departments. Patients who died before initial assessment and those without next of kin to consent were excluded from the study. Initial assessment included haemodynamic status, type and severity of bleeding. In cases of mild bleed and stable patient history details were noted alongwith. In case of heavy bleed, history was taken after the bleeding was controlled. If there were signs of excessive blood loss and/or patient was in a state of shock, steps were taken to stabilize the patient simultaneously with control of epistaxis. Resuscitation was carried out according to Advanced Trauma Life Support (ATLS) principles. After resuscitation all patients underwent a detailed history taking and a through general examination, systemic examination and examination of the nose, throat and ears with special emphasis to identify the site of bleeding. The patients were subjected to investigations of hematological parameters and radiological evaluation. Blood samples were taken and sent for base line haemoglobin estimation and blood grouping and cross matching when indicated. Other relevant investigations were ordered based on clinical suspicion regarding a particular aetiology. The diagnosis of epistaxis was based on clinical history, physical findings, laboratory and radiological investigations with examination under anaesthesia of the nose, nasopharynx and biopsy. All patients were treated conservatively initially and surgical intervention was considered only when conservative means failed to control the epistaxis.

Conservative (non-surgical) treatment included cauterization of the bleeding site using electrocautery, anterior nasal packing and posterior nasal packing. Surgical treatment included resection of intranasal tumors. Arterial ligation and endovascular embolization were not performed as there were no patients with intractable epistaxis. Successful treatment was defined as no recurrent epistaxis following pack removal or no readmission with epistaxis within 24 hours of hospital discharge.

### Data collection, management and Statistical analysis

The data was collected using a pre-tested, structured proforma prepared for the purpose. Data collected included: patient's demographics, cause of epistaxis, anatomical location of bleeding sites, management modalities, need for blood transfusion, length of hospital stay, complications and mortality. The data collected were entered in SPSS version 15.0 for analysis. In descriptive analysis, the mean and standard deviation of continuous variables and percentages of categorical variables were computed

### Ethical consideration

Ethical approval to conduct the study was sought from the WBUCHS/BMC joint institutional ethic review committee before the commencement of the study.

## Results

During the period under study, a total of 104 patients were studied. Eighty-one (77.9%) patients presented through the accident and emergency units and 23 (22.1%) presented in the otorhinolaryngology Clinic. There were 76 males (73.1%) and 28 females (26.9%) with a male to female ratio of 2.7:1. Their ages ranged between 4 and 82 years (mean 32.24 years). The modal age group was 31-40 years. The commonest cause of epistaxis was trauma (30.8%) followed by idiopathic (26.9%) and hypertension (17.3%) (Table 1). All patients with non-traumatic epistaxis had previous history of nasal bleeding ranging from one to five episodes with a mean of three episodes.

**Table 1:** Causes of epistaxis

Causes of epistaxis	Frequency	Percentage
Trauma	32	30.8
Idiopathic	28	26.9
Hypertension	18	17.3
Inflammatory diseases (chronic rhinosinusitis)	6	5.8
Tumors (benign/malignancies)	5	4.8
Iatrogenic	5	4.8
Foreign bodies	4	3.8
Mucosal irritation	3	2.9
Blood dyscrasias	2	1.9
Congenital	1	1.0

Twelve (11.5%) of the patients had more than one cause of the illness. According to the bleeding site, 92 patients (88.5%) had anterior nasal bleeding, 8 (7.7%) had posterior bleeding and the remaining four (3.8%) patients had non-identifiable bleeding sites. The right nasal cavity (62, 59.6%) was more affected than the left (28, 26.9%). Bilateral involvement was recorded in 14 (13.5%) of cases.

Non surgical measures were the main intervention methods in 98.1% of cases. Of this, observation alone without active intervention to arrest bleeding and anterior nasal packing were most common non-surgical measures accounting for 40.4% and 38.5% respectively. Surgical measures mainly tumor resection was carried out in 1.9% of cases (Table 2). Blood transfusion was required in 18 (17.3%) of cases.

**Table 2:** Treatment modalities

Treatment modality	Number of patients	Percentage
Observation alone	42	40.4
Anterior nasal packing	40	38.5
Posterior nasal packing ± Foley catheter balloon	12	11.5
Local cauterization (electrocautery)	8	7.7
Surgical excision of bleeding intranasal tumor	2	1.9
More than one procedure	16	15.4

The overall success rate of treatment was 92.0%. Success rates for various treatment modalities are shown in table 3 below.

**Table 3:** Success rates for various treatment modalities

Treatment modality	Number of patients	Number of patients treated successfully	Success rate (%)
Anterior nasal packing	40	37	92.5
Posterior nasal packing	12	11	91.7
Anterior + posterior nasal packing	13	12	92.3
Local cauterization (electrocautery)	8	7	87.5
Surgical resection of bleeding nasal tumor	2	2	100

Prophylactic broad spectrum antibiotics were prescribed in all patients who had nasal packing, local cauterization and those who underwent surgical resection of intranasal tumors.

The majority of patients 64 (61.5%) were admitted in the ENT wards and the remaining 40 (38.5%) were treated as outpatients. Two (3.1%) patients among the in-patients had severe head injuries and were admitted in the ICU for ventilatory support. Most of in-patients were discharged between 1 day and 7 days after treatment. Six complications were recorded in four patients giving a complication rate of 3.8%. Of these, Hypovolemic shock and recurrent epistaxis were the most common complications accounting for 33.3% each respectively (Table 4).

**Table 4:** Frequency of complications (n = 6)

Complications	Frequency	Percentage
Hypovolemic shock	2	33.3
Recurrent epistaxis	2	33.3
Toxic shock syndrome	1	16.7
Facial edema	1	16.7

The overall mean of duration of hospital stay (LOS) was 7.2 days (range 1 day to 24 days). On the average, patients who have undergone cauterization of the bleeding site required hospitalization for 5.6 days compared to those with anterior nasal packing who had a mean LOS stay of 6.8 days (P < 0.05). Those requiring posterior nasal packing were hospitalized for an average of 10.6 days (P > 0.05).

The majority of patients (90.4%) had good recovery. The details of outcome of patients are shown in table 5

**Table 5:** Outcome of patients

Outcome of patients	Number of patients	Percentage
Good recovery	94	90.4
Discharged on request	2	1.9
Left against medical advice	2	1.9
Referred	1	1.0
Died	5	4.8
Total	104	100

In this study, five patients died giving a mortality rate of 4.8%. The causes of death were; associated severe head injuries in two patients, cardiac arrest during resuscitation, associated tension pneumothorax and nasopharyngeal carcinoma in one patient each respectively.

## Discussion

In this review, epistaxis was found to be more prevalent in the young adults, which is in agreement with Eziyi *et al *[10] but contrary to findings by Pallin *et al *[8] who found a bimodal age-related frequency with peaks among those younger than 10 years and aged 70-79 years. Varshney and Saxena [11] in India reported most of their patients to be older than 40 years which correlates with other reports which showed that epistaxis is a geriatric problem. The low age incidence in our study may be attributed to the fact that the majority of our patients had traumatic epistaxis and patients with traumatic epistaxis tended to be younger than those with atraumatic epistaxis [6-8].

In the present study, epistaxis was found to affect more males than females, with a male to female ratio of 2.7:1. This male preponderance has been documented in literature [10,12-14]. Globally there is a male preponderance in epistaxis except in the geriatric age group in some reports where no significant sex difference exists [11]. The male preponderance in this study may be attributed to high incidence of traumatic epistaxis which tends to affect young males because of their frequent involvement in high risk taking behaviour. Young males are the most active in the population and so are more vulnerable to trauma from nose picking especially among children, fights, road traffic accident with maxillofacial injuries causing epistaxis.

The present study shows that the most common cause of epistaxis was trauma followed by idiopathic and hypertension, which is consistent with other studies in developing countries [10,15,16]. This trauma varied from minor injury such as digital trauma to varying degrees of nasal injury from road traffic injury. The nose being a prominent feature on the face is highly susceptible in craniofacial injury. Most of our patients with epistaxis from trauma were actually victims of road traffic injury. Trauma being the most common cause of epistaxis can partly explain the frequency of this problem in males. This group is the adventurous group in our community. They are often on the road in search of economic well-being thereby making them prone to such accidents. High incidence of traumatic epistaxis resulting from road traffic crashes in our study calls for urgent preventive measures targeting at reducing the occurrence of RTCs in order to reduce the incidence of epistaxis in this region.

Findings in most western literature, cites idiopathic causes as the commonest, followed by trauma [11-14]. In the present study, idiopathic epistaxis was the next most common form of epistaxis after trauma. This is in discordance with what was found in the Eastern part of Nigeria where it represented the dominant form [12].

Hypertension being the third commonest cause in this report shows epistaxis as evidence of poor blood pressure control. This is in keeping with an earlier report from Nigeria of some patients who had epistaxis when their hypertension was not controlled due to cessation of antihypertensive drug therapy [17]. Varsney and Saxena [11] in India recorded hypertension as the second commonest cause of epistaxis after idiopathic causes while Chaiyasate et al [15] in Thailand reported hypertension to be the commonest cause of epistaxis followed by idiopathic causes. The need for regular blood pressure check and compliance to antihypertensive medications must be emphasized.

The management of epistaxis is well summarized in an age-old dictum: resuscitate the patient, establish the bleeding site, stop the bleeding and treat the cause of epistaxis [18]. Dealing with a patient with active severe epistaxis can be bloody. The authors recommend universal precautions for all health care personnel involved in the care of these patients, including face mask with shields, gowns, hair coverage, and double-gloving. The key to controlling most epistaxis is to find the site of the bleeding and cauterizing with silver nitrate or bipolar diathermy [18,19]. The goal of treatment include: hemostasis, short hospital stay, low complication and cost effectiveness of the method of therapy [5,11,18]. Controversy exists concerning the treatment that will best accomplish these goals. Treatment modalities can be separated into two groups; nonsurgical/ion-intervention/conservative and surgical/interventional approaches. Non-surgical approach has been reported to stop the bleeding in more than 80-90% of cases [19]. Anterior nasal packing with gauzed glove finger packing was the most frequent modality of treatment in this study. This form of treatment was reported as an effective treatment in some centers in Nigeria [12], although materials used for the packing vary from center to center. The few patients that had posterior nasal packing were mainly patients with hypertension. Posterior nasal packing was performed using gauze or balloon Foley catheters inserted in the nasopharynx via the nostrils and inflated with sterile water. Anterior nasal packing was used in 38.5% of patients and was successful in 92.5% of them, while posterior nasal packing was successful in 91.7% of the cases where it was tried. Urvashi et al [20] reported successful use of anterior nasal packing in 83.5% case while posterior nasal pack was successful in 95.6% of cases. Nasal packing has the advantage of easy placement and removal; there was no need for an anesthetist or theatre space for that treatment. It is also affordable to the patients. Complications of nasal packing include septal hematoma, sinusitis, syncope during insertion of nasal pack, pressure necrosis of the alae nasi, toxic shock syndrome [20]. Most of our patients did not suffered this due to adequate precautions such as technique of insertion of the pack, use of antibiotics and nasal decongestant were administered as some of the adjunct treatment to forestall this. Only one patient in our study developed toxic shock syndrome. The authors recommend use of prophylactic systemic antibiotics and nasal packing with antibiotic soaked gauze to minimize this complication.

Cautery of the bleeding site can be performed chemically, electrically or with laser [21] though we used only electrical cautery. Cauterization with laser or chemical (Silver nitrate) was not used in our study because of their high costs and lack of availability. Cauterization in the form of electrical cautery was carried out for a group of patients where the bleeding points could be identified during examination. Electrical cauterization was used successfully in 87.5% of cases. This figure was higher than that reported by Urvashi et al [20] in India. Nemer & Mottassim [22] in Jordan reported a success rate of 74.0% which is lower than that of ours. We did not encounter any post cautery complications such as septal perforation or cartilage exposure. Since cauterization of the bleeding point entails a good success rate and no complications it should therefore be the preferred modality of treatment where ever the bleeding site can be visualized. Rigid nasal endoscopy as part of the initial assessment in patients with epistaxis, with direct visualization and control of the bleeding point has been shown to be effective in the majority of patients, reducing the need for nasal packing [18].

In this study, surgical treatment was done only in 1.9% of patients who presented with bleeding intranasal tumor and it was successful in 100% of them. Similar finding was also reported in Nigeria [17]. No surgical ligation of any vessel or endovascular embolization was carried out on any patient in this study. Arterial ligation and embolization of feeding vessels are the last resort for intractable epistaxis [23]. Selection of the artery depends upon the area of the nasal cavity whether upper or lower half or angiographic findings. Choice is usually between anterior ethmoidal artery or internal maxillary artery through an external approach. However, Sphenopalatine artery, termination of internal maxillary artery, may be ligated endosmotically [24,25]. Embolization of feeding vessels may be an option in these cases, but carries high risk of complications [26]. The risks of surgical treatment include the risk of anaesthesia, blindness, oro-antral fistula, ophthalmoplegia, cosmetic deformity, infra orbital nerve dysfunction. These complications were not observed in our study.

The rate of blood transfusion for epistaxis has been reported in literature to range between 6.92 - 15.1% which is less than our blood transfusion rate in our study [11,14]. This high rate of blood transfusion is probably due to severe acute blood loss from the trauma sustained.

The use of antimicrobial prophylaxis in the presence of nasal packing for the treatment epistaxis remains controversial [18,27]. Most of literatures recommend that patients with high risk nasal packing should be started on prophylactic antibiotics, due to an increased risk for sinusitis and toxic shock syndrome. Blood soaked pack and raw mucosal surface are good media for bacterial multiplication resulting in infection including sinusitis and sometimes toxic shock syndromes [28].

The mean length of hospital stay in our stay was 7.2 days which is higher than that reported by other authors [10,22]. Patients who underwent local cauterization were found to have significant shorter LOS compared to those with anterior nasal packing. Those requiring posterior nasal packing remained in hospital for an average of 11.6 days which is higher compared to those with local cauterization or anterior nasal packing. From our observations of average hospital stay with different treatment modalities, we are able to infer that cauterization of the bleeding point reduces hospital stay as compared to anterior nasal packing. However, the difference was not significant comparing anterior nasal packing and posterior nasal packing. Availability of nasal endoscopes which offers both proper visualization and direct facility for endoscopic cauterization to the area that is not easily accessible may have been able to further reduce the hospital stay and the discomfort of postnasal packing.

Our mortality rate in the present study was found to be high than that reported in other studies [10,16,17]. The factors responsible for this finding in our study were; associated severe head injuries, cardiac arrest associated tension pneumothorax and nasopharyngeal cancer.

## Conclusion

Trauma resulting from road traffic crush (RTC) remains the most common etiological factor for epistaxis in our setting. Most cases were successfully managed with conservative (non-surgical) treatment alone such as nasal packing and local cauterization. Non-surgical treatment is still useful to arrest nasal bleeding and it is safe and cost-effective, and surgical intervention should be the last resort. Reducing the incidence of trauma from RTC will reduce the incidence of emergency epistaxis in our centre.

## Competing interests

The authors declare that they have no competing interests.

## Authors' contributions

JMG designed the study, contributed in literature search, data analysis, manuscript writing & editing. PLC participated in study design, data analysis, manuscript writing, editing and submission of the manuscript. All the authors read and approved the final manuscript.

## Authors' information

JMG: Senior Consultant General/ENT surgeon, Senior Lecturer and Head, Department of Surgery, Well Bugando University Collage of Health Sciences.PLC: Consultant general surgeon and Lecturer, Department of Surgery, Well Bugando University Collage of Health Sciences

## Pre-publication history

The pre-publication history for this paper can be accessed here:

http://www.biomedcentral.com/1472-6815/11/8/prepub
